# Preparedness for influenza vaccination during a pandemic in the World Health Organization Western Pacific Region

**DOI:** 10.5365/wpsar.2018.9.5.001

**Published:** 2018-12-20

**Authors:** Leila Bell, Lisa Peters, James D Heffelfinger, Sheena G Sullivan, Alba Vilajeliu, Jinho Shin, Joseph Bresee, Erica Dueger

**Affiliations:** a WHO Regional Office for the Western Pacific, Emerging Diseases Surveillance and Response.; b WHO Collaborating Centre for Reference and Research on Influenza at the Peter Doherty Institute for Infection and Immunity, Melbourne, Victoria, Australia.; c Centre for Epidemiology and Biostatistics, School of Population and Global Health, University of Melbourne, Melbourne, Victoria, Australia.; d Centers for Disease Control and Prevention, Atlanta, Georgia.

## Background

Influenza vaccination is a key public health intervention for pandemic influenza as it can limit the burden of disease, especially in high-risk groups, minimize social disruption and reduce economic impact. (*1*) In the event of an influenza pandemic, large-scale production, distribution and administration of pandemic vaccines in the shortest time possible is required. In addition, monitoring vaccine effectiveness, coverage and adverse events following immunization (AEFI) is important. Since seasonal influenza vaccination programmes require annual planning in each of these areas, establishing and strengthening annual influenza programmes will contribute to pandemic preparedness. (*2*) This paper presents efforts made in the World Health Organization (WHO) Western Pacific Region to improve seasonal influenza vaccination and pandemic preparedness.

Several initiatives have been established in response to the World Health Assembly goal set in 2003 of reaching 75% influenza vaccination in persons ≥ 65 years by 2010. (*3*) In 2006, the Global Action Plan for Influenza Vaccines (GAP) (2006–16 strategy) aimed to increase the use of seasonal influenza vaccines, increase vaccine production capacity and promote research and development for improved vaccines and vaccine production technologies. (*3*) The goal of GAP was to produce enough vaccine to immunize 70% of the global population with two doses of the influenza vaccine within six months of the identification of a pandemic strain (approximately 10 billion doses) and to develop national vaccine deployment plans for pandemic influenza.

The 2009 pandemic highlighted that there was a lack of existing national influenza vaccination programmes, which was a barrier to rapid deployment of pandemic vaccines. The primary challenges in the WHO Western Pacific Region for vaccination during a pandemic response was the limited experience in many countries in conducting vaccination campaigns, mobilizing financial support for vaccine deployment, refining national planning guidelines and deployment plans and establishing sufficient cold-chain capacities. (*4*)

In 2011, the World Health Assembly adopted the Pandemic Influenza Preparedness (PIP) framework to address more predictable, efficient and equitable access to vaccines and medicines during future pandemics through establishing antiviral and interpandemic vaccine stockpiles. (*5*) In 2012, the Partnership for Influenza Vaccine Introduction (PIVI) (*6*) – a collaboration between the Global Health Task Force, the United States Centers for Disease Control and Prevention, various ministries of health and pharmaceutical and technology industry partners – also supported increased pandemic readiness by expanding national seasonal influenza vaccination programmes in several countries in the Region, including the Lao People's Democratic Republic, Mongolia and Viet Nam.

### Production of influenza vaccines

The process and logistics required to manufacture and produce seasonal influenza vaccines can be used for possible pandemic strains when quick action is required on a large scale. (*2*) The capacity for an effective and timely pandemic vaccine response remains limited by the time required to manufacture pandemic vaccines and by global vaccine production capacity. (*7*) Strong systems for detection of new influenza variants are also critical. The Global Influenza Surveillance and Response System is tasked with monitoring influenza strains to detect new variants through a network of laboratories around the world. (*8*) To ensure adequate production for influenza vaccines during a pandemic, multiple influenza vaccine manufacturers are required so that supply meets demand, vaccine pricing is competitive and manufacturers with capacity and operational plans in place can switch from seasonal to pandemic influenza vaccine production as needed.

In the Western Pacific Region, four countries produce influenza vaccines with three (Australia, China and Republic of Korea) distributing WHO-prequalified influenza vaccines globally. Two (Japan and Republic of Korea) recently built large-scale, cell-based manufacturing plants. Efforts are ongoing to strengthen influenza vaccine supply hubs in Asia and the Pacific, focusing on GAP grantee manufacturers in China, India, Thailand and Viet Nam. (*9*)

### Influenza vaccine regulatory approval and deployment plans

Country vaccination programmes will need policies and effective regulatory pathways in place to rapidly accept, distribute and administer the new pandemic vaccine. Effective mechanisms for seasonal influenza vaccination distribution can be used for distribution during a pandemic. (*2*, *10*) WHO encourages optimizing regulatory pathways and the inclusion of a vaccine deployment plan when developing or updating a national pandemic preparedness plan.

An example, albeit on a small scale, of using established seasonal influenza detection, reporting and distribution mechanisms to respond to unusual influenza activity occurred in May 2016 when four pregnant women with severe acute respiratory infection died in Fiji within a five-week period. The laboratory detected an apparent variant of influenza A(H1N1)pdm09 from specimens isolated from two cases. (*11*) As per testing protocols, the isolates were sent to a WHO Collaborating Centre to confirm that the virus was truly a variant. At the same time, 150 courses of oseltamivir and 20 000 adult seasonal influenza vaccine doses were distributed, targeting pregnant women and health-care workers. The Collaborating Centre determined that neither of the two A(H1N1)pdm09 isolates were novel variants (internal communication). This strong detection and reporting system, aligned with a global response system able to verify laboratory results and assure timely delivery of vaccine and oseltamivir, resulted in an appropriate response to the event. However, had this been a new influenza strain, additional efforts to develop a new vaccine would have been required.

### Influenza vaccine policy development and seasonal influenza vaccination programme implementation

Over the past decade, the number of countries and areas with seasonal influenza immunization policies has increased, as has the number of vaccines distributed globally. (*12*, *13*) In the Western Pacific Region, the number of Member States that reported having influenza immunization policies increased from 12 in 2011 to 16 in 2014. (*13*, *14*) Based on a survey conducted in the Region in 2017, 24 of the 37 countries and areas reported having an influenza immunization policy targeting at least one of the WHO-recommended priority groups (WHO, unpublished data, 2017). However, evidence has also indicated that formal policies or recommendations do not necessarily lead to wider distribution of influenza vaccine as influenza vaccine distribution by pharmaceutical companies per 1000 population decreased between 2011 and 2014. (*13*, *15*) Distribution data also cannot account for vaccine wastage or returns and do not provide information on implementation or vaccination rates in high-risk groups. In the Western Pacific Region, improving seasonal influenza vaccination coverage is challenging due to extensive geographic and demographic diversity and varied influenza transmission patterns, burden and vaccination policies. (*14*, *16*)

The Lao People's Democratic Republic, one of the countries that receives PIP support and the first country to receive support from PIVI, provides an example of using influenza surveillance data to improve vaccination coverage. As their influenza surveillance data indicated a substantial disease burden, they developed a multiyear introduction plan for influenza vaccine, established systems to evaluate the vaccine programme and are developing a sustainability plan. (*17*) Since 2014, more than 1.5 million persons have been vaccinated with a focus on high-risk groups such as pregnant women and health care workers. (*6*) The Lao People's Democratic Republic has developed a robust vaccination programme to support timely and efficient vaccine use in response to the next influenza pandemic.

Viet Nam and Mongolia are also working to strengthen their influenza programmes through several initiatives including strengthening the National Immunization Technical Advisory Group and conducting Knowledge, Attitudes and Perceptions surveys to inform their influenza vaccine communication strategies. Viet Nam trained and vaccinated nearly 11 000 health-care workers in 2017, and Mongolia conducted a national survey on AEFI of health-care workers and pregnant women who received the influenza vaccine. (*6*) These efforts aim to create sustainable seasonal influenza programmes by training health-care workers, developing communication materials, improving vaccine acceptability, establishing monitoring systems for AEFI and assessing influenza vaccine coverage and impact.

## Conclusions

The Western Pacific Region has made improvements to its seasonal influenza vaccination programmes and vaccination planning for pandemic preparedness. This includes improved laboratory capacity to rapidly identify new circulating virus strains, support for development of influenza vaccine regional supply hubs, capacity-building for national regulatory processes and development of vaccine deployments plans. Efforts are also ongoing to strengthen influenza surveillance systems to determine disease severity to inform the priority groups to target when designing influenza vaccine policies. Continued political commitment from Member States and support by the global community are needed to ensure that sustainable and robust national seasonal influenza programmes are in place for effective response to the next pandemic.
